# Kinetics of MSC-based enzyme therapy for immunoregulation

**DOI:** 10.1186/s12967-019-2000-6

**Published:** 2019-08-13

**Authors:** Alexandra Burr, Biju Parekkadan

**Affiliations:** 1 Department of Biomedical Engineering, Rutgers University, Piscataway, NJ 08854 USA; 2 Department of Medicine, Rutgers Biomedical and Health Sciences, Piscataway, NJ 08854 USA; 3 Department of Surgery, Center for Surgery, Innovation & Bioengineering, Massachusetts General Hospital, Harvard Medical School and the Shriners Hospitals for Children, Boston, MA 02114 USA; 4 Harvard Stem Cell Institute, Cambridge, MA 02138 USA

**Keywords:** Mesenchymal stem cells, ATP, Purinergic, Immunomodulation, Inflammation, Pharmacokinetics, Immune model, Autoimmune disease, MSC, Hydrolysis, Lymphocyte, Monocyte, Cell therapy

## Abstract

**Background:**

Mesenchymal stromal cells (MSC) demonstrate innate and regulatory immunologic functions and have been widely explored for cell therapy applications. Mechanisms by which MSCs achieve therapeutic effects are theorized, though appropriate dosing and duration of these mechanisms in vivo warrant deeper investigation. One, rapid immunosuppressive function of MSCs is through ectoenzyme expression of CD73 and CD39 which cooperatively hydrolyze inflammatory, extracellular adenosine triphosphate (ATP) to anti-inflammatory adenosine. Extracellular ATP has a key role in autoimmune and inflammatory diseases, which administered MSCs have the potential to modulate in a timescale that is befitting of shorter acting therapeutic function.

**Methods:**

In vitro experiments were performed to determine the hydrolysis rates of ATP by MSCs. Through kinetic modeling from experimental results, the rate at which a single cell can metabolize ATP was determined. Based on these rates, the ability of MSCs to downregulate inflammatory signaling pathways was prospectively validated using model system parameters with respect to two different mechanisms: extracellular ATP stimulates lymphocytes to suppress proliferation and induce apoptosis and with co-stimulation, it stimulates monocytes to release pro-inflammatory IL-1β. MSCs were co-cultured with immune cells using transwell inserts and compared to immune cell only groups.

**Results:**

Hydrolysis of ATP was efficiently modeled by first-order enzyme kinetics. For in vitro culture, the rate at which a single cell can hydrolyize ATP is 8.9 nmol/min. In the presence of extracellular ATP, cocultures of MSCs reduced cytotoxicity and allows for proliferation of lymphocytes while limiting IL-1β secretion from monocytes.

**Conclusions:**

Such use of these models may allow for better dosing predictions for MSCs to be used therapeutically for chronic inflammatory diseases such as rheumatoid arthritis, diabetes, pancreatitis, and other systemic inflammatory response syndromes. For the first time, the effect of MSCs on ATP hydrolysis in immune cell response is quantitatively analyzed on a cell-molecule basis by modeling the hydrolysis as an enzyme–substrate reaction. The results also give insight into MSCs’ dynamic response mechanisms to ameliorate effects of extracellular ATP whether it be through positive or negative regulation.

**Electronic supplementary material:**

The online version of this article (10.1186/s12967-019-2000-6) contains supplementary material, which is available to authorized users.

## Background

Adenosine triphosphate (ATP) is found at high concentrations intracellularly, which range from 3 to 10 mM, but when concentrations of extracellular ATP are high, this often serves as a warning signal [1]. High concentrations of ATP signal inflammatory pathway activation which can lead to necrosis, apoptosis or secretion of proinflammatory cytokines [2]. Release of ATP can be due to cell lysis, apoptosis, or from live cells through pannexin-1 channels by chemical, mechanical or paracrine signals [3]. This release plays a role in both pathology and exacerbation of chronic inflammatory diseases such as osteoarthritis, rheumatoid arthritis, irritable bowel syndrome, emphysema, COPD and Muck–Wells syndrome [4–6].

The effects of ATP on immune cells varies greatly by cell type depending on indication. When CD3+/CD28− T-lymphocytes are exposed to high extracellular ATP concentrations, it has been shown that subset populations respond in different ways: T-regulatory cells are activated and proliferate while apoptosis is induced in CD4+ T-cells [7]. Additionally, when co-stimulated with lipopolysaccharide solution (LPS), monocytes and macrophages are activated by high concentrations of ATP to secrete pro-inflammatory IL-1β [8]. The immune system has innate mechanisms to recognize and respond to extracellular ATP and control inflammasome activation. Purinergic receptors respond to extracellular ATP depending on the concentration where responses to low concentrations are mediated by P2R, intermediate concentrations by P2X1, P2Y2 and others, and very high concentrations above 100 μM are mediated by P2X7 [9]. They allow the cell to mediate a response to ATP, while ectoenzymes ectonucleoside triphosphate diphosphohydrolase-1 (CD39) and ecto-5′-nucleotidase (CD73) hydrolyze ATP to AMP and anti-inflammatory adenosine, respectively. The normal extracellular concentrations of ATP are in the 10 nM range, which is a millionth of what can occur in chronic inflammation. The level of extracellular ATP required for activation of purinergic receptors is around 500 nM, but after extended periods of being exposed to these high concentrations, purinergic enzymes have a lessened effect [10].

Many indications for which extracellular ATP is present and inducing these immune responses are multi-faceted diseases that require complex treatments. One pharmaceutical treatment method used currently aims to block the P2X receptors which disable the cell from responding, and therefore activating downstream inflammatory pathways. For rheumatoid arthritis, AstraZeneca developed the AZD9056 treatment which is an antagonist for P2X receptors [11]. However, this may be limited by the number of receptors it can block, while the extracellular ATP levels remain high.

In the case of cell-based therapies, mesenchymal stromal cells (MSCs) have been widely explored for their immunomodulatory functions. They have shown promise for osteoarthritis by administering bone marrow MSCs intra-articularly with hyaluronic acid [12]. Systemic injection therapies are also being developed which allow MSC localization in the lungs and spleen to treat patients with autoimmune-associated tissue damage such as multiple sclerosis, polymyositis, atopic dermatitis, and rheumatoid arthritis [13]. MSCs possess intrinsic mechanisms to modulate inflammatory environments, secrete anti-inflammatory soluble factors, and home to injured areas, making them a multi-faceted therapy for treating complex diseases [13–15].

**Fig. 1:**
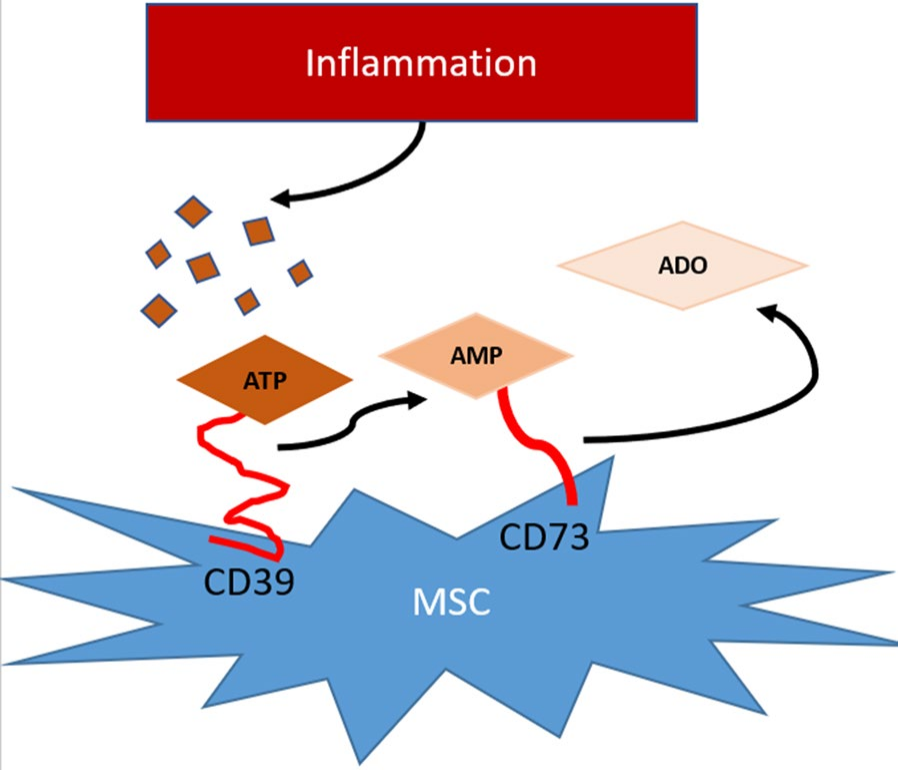
MSCs express extracellular ectoenzymes to hydrolyze ATP to adenosine. ATP is converted to adenosine by two sequential enzymes, CD39 and CD73. It has been shown that MSCs express both enzymes and have the potential to modulate the expression of these enzymes on other cell types

Looking specifically at ATP, MSCs are shown to express both ectoenzymes, CD39 and CD73 required for hydrolyzing ATP to adenosine, as shown in Fig. 1 [16–18]. When co-cultured with immune cells, MSCs can reduce proliferation and apoptosis of T-lymphocytes in the presence of stimulants but have not been explored for responses caused by ATP stimulation [18, 19]. Additionally, it has been shown that coculturing immune cells with MSCs can alter the expression of CD39 and CD73. MSCs upregulate CD39 expression in the presence of activated T-cells, monocytes up-regulate CD73 and natural killer cells acquire CD73 expression in the presence of MSCs [20–22]. This shows that MSCs may not only work to directly reduce inflammation caused by extracellular ATP but help cells to self-regulate as well. However, the dose at which MSCs are needed to have therapeutic efficacy is often characterized based on in vivo results and by typically low persistence once administered [23]. The hydrolysis of extracellular ATP could be a rapid therapeutic mechanism that has immunomodulatory activity within the timescales of MSC persistence.

Here, we explore MSCs ability to hydrolyze ATP to anti-inflammatory adenosine in a quantitative way to explain their response more deeply than just positive or negative. Furthermore, we analyzed the physiologic response of different immune cell types to better understand the effect of extracellular ATP and MSCs as immunomodulators. We demonstrate that ATP hydrolysis to adenosine by MSCs follows first-order enzyme kinetics. The experimentally-derived rates were used to hypothesize physiological responses in immune cells. Co-cultures of MSCs showed that at predicted doses, MSCs can restore proliferation, and prevent apoptosis in lymphocytes as well as suppress monocyte secretion of IL-1β.

## Methods

### Cell culture

Human peripheral blood mononuclear cells (PBMCs) were obtained from the New York Blood Center as leukopacks, following Institutional Review Board (IRB) guidelines. 35 mL aliquots of whole blood were layered on top of 15 mL Ficoll solution (Sigma-Aldrich St. Louis, MO) and centrifuged at 400*g* for 60 min at 4 °C. PBMCs were collected from the top layer and cryopreserved in 90% Gibco fetal bovine serum (FBS) and 5% dimethyl sulfoxide. Batch verification was performed for each donor to ensure viability, stimulation capability and proliferation. For lymphocyte experiments, PBMCs were thawed and seeded in 24 well plates at 4 million cells/mL of complete RPMI 1640 medium (Fisher Scientific, Waltham, MA), 10% Gibco FBS (ThermoFisher Waltham, MA) and 1% penicillin–streptomycin. For monocyte experiments, PBMCs were thawed and seeded in 24 well plates (Corning, Corning NY) at 3 million cells/mL of complete RPMI 1640 medium, 6% human plasma and 1% penicillin–streptomycin. Monocytes adhered overnight and the following day, the wells were rinsed 3 times with phosphate buffered saline (PBS) to remove non-adherent cells.

Whole bone-marrow was processed according to manufacturer’s protocol to isolate mesenchymal stromal cells (Lonza, Allendale, NJ). Bone-marrow derived mesenchymal stromal cells (MSCs) were cultured in complete media composed of alpha-MEM (ThermoFisher Waltham, MA), 10% Hyclone FBS (GE Life Sciences Pittsburgh, PA), 1% penicillin–streptomycin (ThermoFisher, Waltham, MA), and 2.5 μg/L human basic fibroblast growth factor (Waisman Biomanufacturing, Madison WI). Before experiments, cells were allowed to adhere overnight. Media was changed every other day. For coculture experiments, MSCs were seeded at 50,000 cells per transwell insert for a 24-well plate (Corning, Corning NY) and allowed to adhere overnight in 0.7 mL complete medium.

### Characterization of extracellular enzymes

FITC-conjugated CD39 and APC-conjugated CD73 antibodies were used to characterize extracellular expression of purinergic enzymes (BD Biosciences, San Jose, CA). Adherent cells were detached with Trypsin (ThermoFisher, Waltham, MA) and PBMCs were centrifuged for 5 min at 500*g*. The monoclonal antibodies were incubated in 1 mL cell suspension for 10 min protected from light and rinsed 2 times with 2% bovine serum albumin in PBS. Flow cytometry was performed using BD FACS Canto II and FACS Diva software (BD Biosciences, San Jose, CA). Data was analyzed using FlowJo software (Tree Star, Ashland, OR). Positive staining was gated based on negative control cells that do not express CD39 or CD73.

### ATP hydrolysis experiments

To expose cells to extracellular ATP, adenosine 5′-triphosphate disodium salt hydrate (Sigma-Aldrich, St. Louis, MO) was reconstituted in sterile, distilled water at a concentration of 50 mM, aliquoted and stored at – 20 °C. Adherent cells were seeded in various well plates at a density of 25,000 cells/cm^2^ and allowed to adhere overnight. Well plate sizes used were 6, 12, 24, 48 and 96-well. The following day, exposure media was prepared by diluting the ATP solution in media at concentrations ranging from 16 to 560 µM (nmol/mL). Culture media was aspirated and replaced with exposure media.

Time 0 began when soluble ATP containing-media was added to the cells. Then, after each hour of exposure, 10 µL from each well was sampled and transferred to a 96-well plate. A luciferase reagent was optimized to contain 10 mM d-Luciferin substrate (Fisher Scientific, Waltham, MA), 14 mg/mL Renilla Luciferase (Sigma-Aldrich, St. Louis, MO), 100 mM magnesium chloride (Sigma-Aldrich, St. Louis, MO), and sterile, distilled water. 95 µL of the luciferase reagent was added to each well of the 96-well plate, protected from light and shaken for 10 s, before being read using the VarioSkan plate reader (ThermoFisher, Waltham, MA) with luminescence parameters and ATP standards and controls. Because of the short half-life of ATP, standards were used at each timepoints and used to convert relative light units to nmol/mL of ATP. The amount hydrolyzed in each well was calculated by subtracting the remaining amount in individual wells at each timepoint from the initial concentration.

To determine the amount of adenosine being produced as a product of ATP hydrolysis, a fluorometric adenosine assay kit was used according to manufacturer’s protocol (Abcam, Cambridge, UK). In brief, 50,000 MSCs were seeded in a 24-well plate and allowed to adhere overnight. Cells were dosed with 500 nmol of ATP and supernatant was sampled starting after 30 min to 2.5 h of exposure and assayed in duplicate for adenosine concentration.

To correlate ATP hydrolysis with CD39 and CD73 enzymatic activity, ENTPD-1&2 inhibitor POM-1 (Tocris Bio, Bristol, UK) was used at a concentration of 100 µM. Cells were treated for 20 min with POM-1 solution and was removed before ATP dosing. Inhibition of hydrolysis was measured using the adenosine assay rather than ATP assay, as POM-1 is known to interfere with luciferase-based ATP assays [24].

### Non-linear regression models

Using the ATP hydrolysis over time, the total amount hydrolyzed after 90 min was used to determine the rate of hydrolysis for different substrate concentrations. Using non-linear regression fit in Matlab, data was fit to a Michaelis Menton model for each of the population sizes to determine the maximum hydrolysis rate (Vmax) and substrate concentration at half the maximum (Km) when cells were seeded in 6, 12, 24, 48 and 96-well plates. The Vmax values were plotted as a function of the normalized cell population size (*mL/cm^2^) and fit with a linear curve.

### Lymphocyte experiments

PBMCs were thawed and stained with 2.5 µM CFSE (ThermoFisher, Waltham, MA), a cell permeable fluorescent staining dye, which dilutes in intensity as cells proliferate, at 2 million cells/mL for 5 min protected from light. After 2 PBS rinses, cells were seeded in 24-well plates with a total volume of 0.6 mL complete media and cultured overnight in 24-well plates at 2 million cells/mL. The following day, they were stimulated with 500 ng/mL and 250 ng/mL of anti-CD3 and anti-CD28 monoclonal antibodies respectively (Fisher Scientific, Waltham, MA), and a range of soluble ATP from 0.5 to 6 mM.

For co-culture wells, 50,000 MSCs were allowed to adhere overnight in transwell inserts and added to the PBMC cultures at the time of stimulation with anti-CD3/CD28 and ATP. After 8 h, the inserts were removed, and anti-CD3/CD28 stimulation of the PBMCs continued.

96 h after stimulation cells were centrifuged and dead cells were stained with ethidium homodimer-1 (Fisher Scientific, Waltham, MA). A control well for dead cells was used by exposing PBMCs to 50% ethanol solution for 10 min before staining. After 2 PBS rinses, cells were resuspended in 2% bovine serum albumin in PBS and stored on ice. All flow cytometry was performed using the BD Biosciences Canto II. An unstimulated control was used to gate non-proliferative cells and proliferation was determined by a shift from the control as a percentage of cells. A negative control of HEK293 cells were seeded in transwell inserts at the same density of MSCs to show that effects were due to purinergic enzyme expression. Analysis was done in FlowJo and lymphocytes were gated using forward and side scatter plots. Dead cells were gated based on the cytotoxic control.

### Monocyte experiments

Monocytes adhered overnight and the following day, cells were stimulated with 1× lipopolysaccharide solution (LPS) (ThermoFisher, Waltham, MA) for 4 h. Soluble ATP ranging from 0.5 to 3 mM was then added to wells for an additional 2 h. For co-culture wells, MSCs in transwell inserts were added to the monocyte cultures at the time of LPS stimulation at a density of 50,000 cells per transwell. After 2 h of ATP stimulation, inserts were removed, and the supernatant was collected and stored at – 20 °C until analysis was performed.

To measure the IL-1beta secretion, a sandwich ELISA was performed according to manufacturer protocols (R&D Systems, Minneapolis, MN). 100 μL of sample was added at 1:1, 1:10 and 1:50 dilutions in duplicates and a standard curve fit to a 4-parameter logistic curve was used to determine the concentration of IL-1β in each well.

### Statistics

All experiments were done in at least triplicate (n = 3) and assay duplicates were used in plate reader experiments such as luminescence and ELISA. For kinetic modeling, least squares regression was used to determine the correlation of fit. For the proliferation experiment, statistical significance was determined using a one-way ANOVA with Tukey’s post hoc analysis. Significance was denoted for p < 0.10 and p < 0.05. To determine statistical significance between monocyte IL-1β secretion, a one-tailed, unpaired t-test was used with significance p < 0.10. All errors bars are represented as ± SD from the mean.

## Results

### MSCs express the ectoenzymes CD39 and CD73

**Fig. 2:**
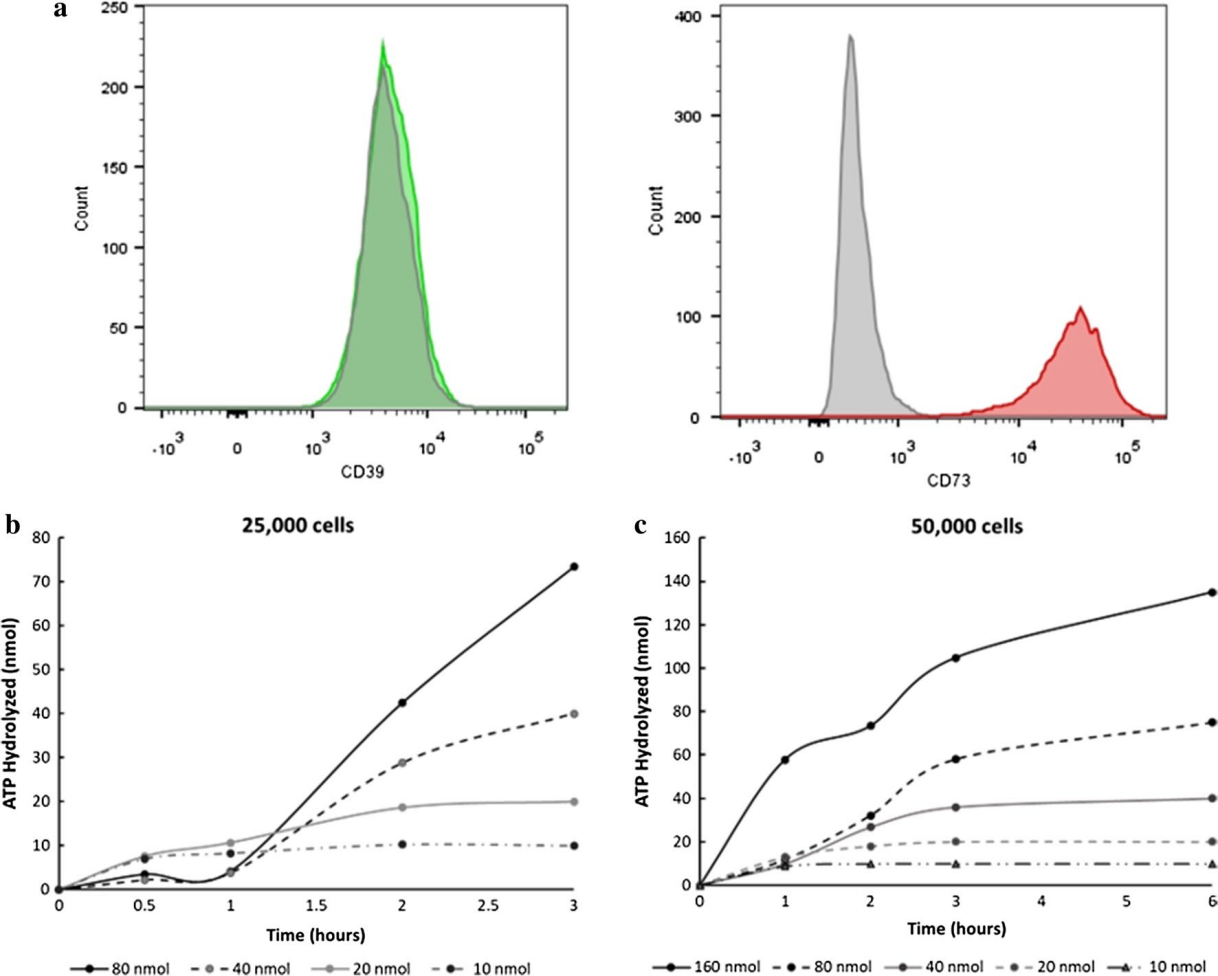
Ectoenzyme expression and MSC hydrolysis of ATP over time. **a** Fluorescently conjugated antibodies against CD73 and CD39 were used to determine MSC expression levels. CD73 is highly expressed by MSCs, while CD39 is lowly expressed. **b**, **c** When exposed to extracellular concentrations of ATP, MSCs hydrolyze it at rates that depend on the dose exposed as well as how many cells are in the well. Increasing dose exposure increases the rate of hydrolysis and increases the cell population size, increasing the total amount of ATP that can be hydrolyzed within a given time. Hydrolysis was measured for every population size ranging from a 96-well to a 6-well plate and example population hydrolysis rates for 25,000 cells in a 48-well plate (**b**) and 50,000 cells in a 24-well plate (**c**) are shown, where n = 3 for each group

It has been shown that CD39 and CD73 enzymes are expressed by MSCs and PBMCs, but because these are primary cells, the expression may be batch-dependent and vary in expression level. As shown in Fig. 2a, MSCs highly express CD73 while CD39 is lowly expressed. The level at which these enzymes are expressed has been shown to vary with ATP exposure as well, and here we show that in the experiments carried out, the low levels of CD39 expression either increased when exposed to high levels of ATP or were sufficient in hydrolyzing the amount exposed. Because CD39 is the first sequential step in the reaction and the assay measured the amount of ATP remaining in the well, we can assume what has been hydrolyzed at each timepoint has also been converted to adenosine by abundantly expressed CD73.

### MSCS hydrolyze ATP in a substrate dependent manner which follows Michaelis Menton kinetics

As the population size increased, the amount of ATP hydrolyzed by early timepoints also increased and therefore, the range of exposure was adjusted for each group to ensure around 100% hydrolysis within the timespan appropriate for the assay (Fig. 2b, c). The high range of exposure for 25,000 MSCs to hydrolyze is around 80 nmols while it is around 160 nmols for 50,000 cells. For all doses, the rate at which the cells hydrolyze ATP is dose-dependent, which can be seen by the increase in slope in Fig. 2c from 10 to 160 nmol exposure. As expected with the abundant expression of CD73, the levels of adenosine produced as a result of enzyme activity were similar to the amounts of ATP hydrolyzed and increased with time after ATP dosing (Additional file 1: Figure S1A). Levels of adenosine show around 95% conversion of AMP to adenosine by producing 112 nmol adenosine out of 118 nmol ATP measured after 2.5 h. Additionally, the hydrolysis by MSCs can be primarily attributed to CD39 and CD73 activity and by inhibiting CD39 activity with POM-1, there is little to no adenosine produced (Additional file 1: Figure S1B). Interestingly, many fibroblast cell types also express these ectoenzymes, including cancerous fibroblasts, which demonstrates in part how these cells are able to evade immune response: by hydrolyzing ATP to release anti-inflammatory adenosine. We show hydrolysis over time for these cell types is comparable to that of MSCs (Additional file 1: Figure S2).

**Fig. 3:**
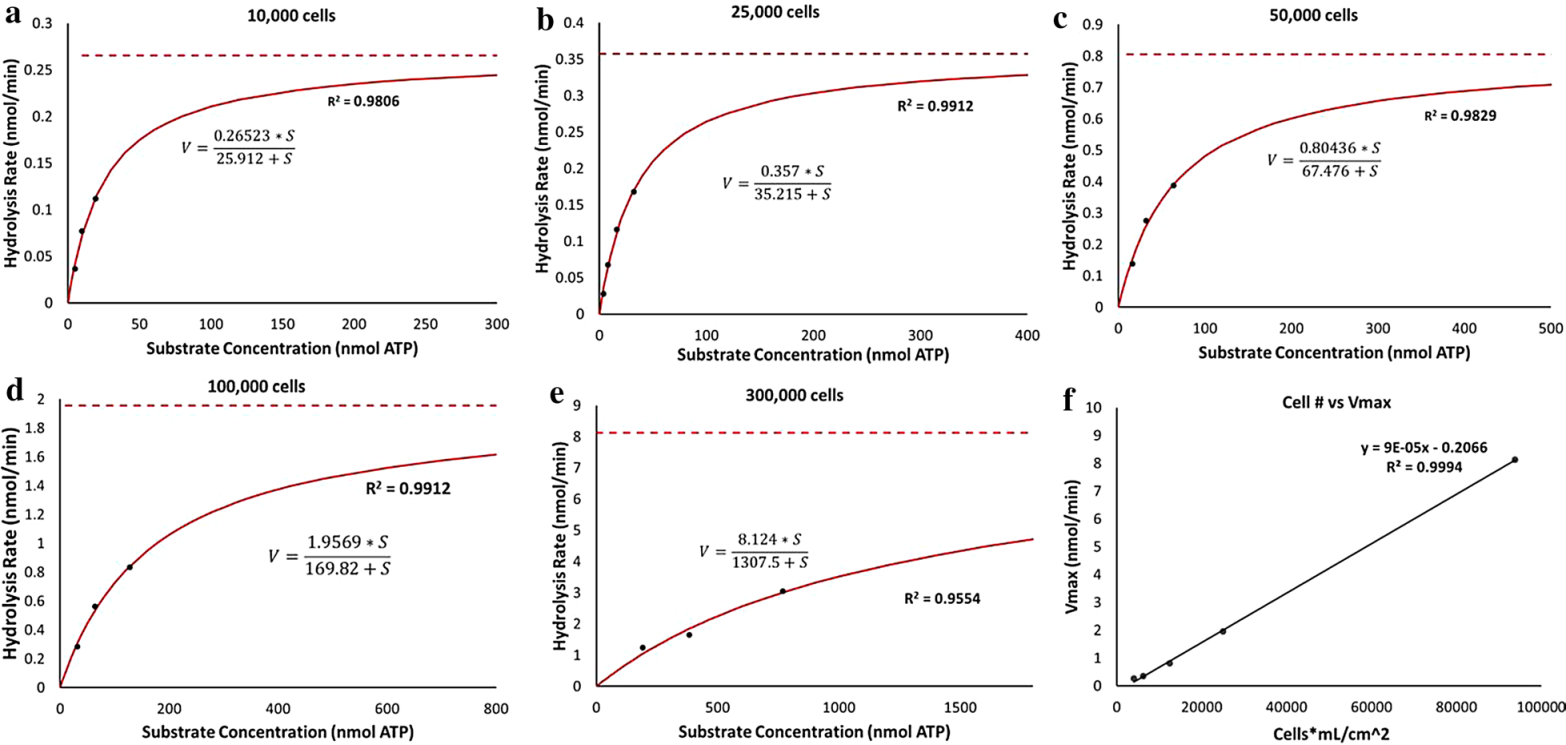
Michaelis Menton modeling of MSC hydrolysis. **a**–**e** Rates for each population size were determined as a function of dose by finding the slopes of hydrolysis against time graphs. Using non-linear regression fitting, rates as a function of dose were fit to first-order enzyme kinetic equations, which show that Vmax and Km increase as population size increases. For all models, R^2^ > 0.95 and n = 3. **f** There is a linear relationship between Vmax and normalized cell population, of which the slope determines the maximum hydrolysis rate per cell

We hypothesized that MSC hydrolysis must follow enzyme–substrate kinetics, however we expected it follow a more complex kinetic profile than first-order systems. Surprisingly, we show that fitting the experimental data to Michaelis Menton models where the hydrolysis rate (V) is graphed as a function of the ATP dose (substrate), achieves high correlation of fit (R^2^ > 0.95) for all population sizes tested (Fig. 3a–e). For 10,000 cells/well in a 96-well plate the Vmax predicted by the model is 0.26 nmol ATP/min (Fig. 3a) while for 300,000 cells in a 6-well plate, the Vmax predicted is 8.1 nmol ATP/min (Fig. 3e).

### Maximum hydrolysis rate is linearly correlated to the normalized cell population size

To test the predictive power of the model, the hypothetical maximum hydrolysis rate (Vmax) obtained from the graphs for each cell population size was plotted against the normalized population size. As shown in Fig. 3f, the Vmax values predicted provide a linear prediction for increasing with cell population size, where R^2^ > 0.99. For predictive testing, the slope of the graph can be used to estimate the amount of ATP to be hydrolyzed by a single MSC/min, which was calculated to be 90 fmoles/min or 5.4 pmol/h when operating at the Vmax. In 2D model systems, the correlation shows that scale-out dosing is applicable. This would mean that for 50,000 MSCs in a 24-well plate transwell insert, the maximum amount of ATP that can be hydrolyzed per hour is 71 nmol when operating at the Vmax.

### MSCs allowed for lymphocyte proliferation when stimulated with anti-CD3/CD28 and ATP

**Fig. 4:**
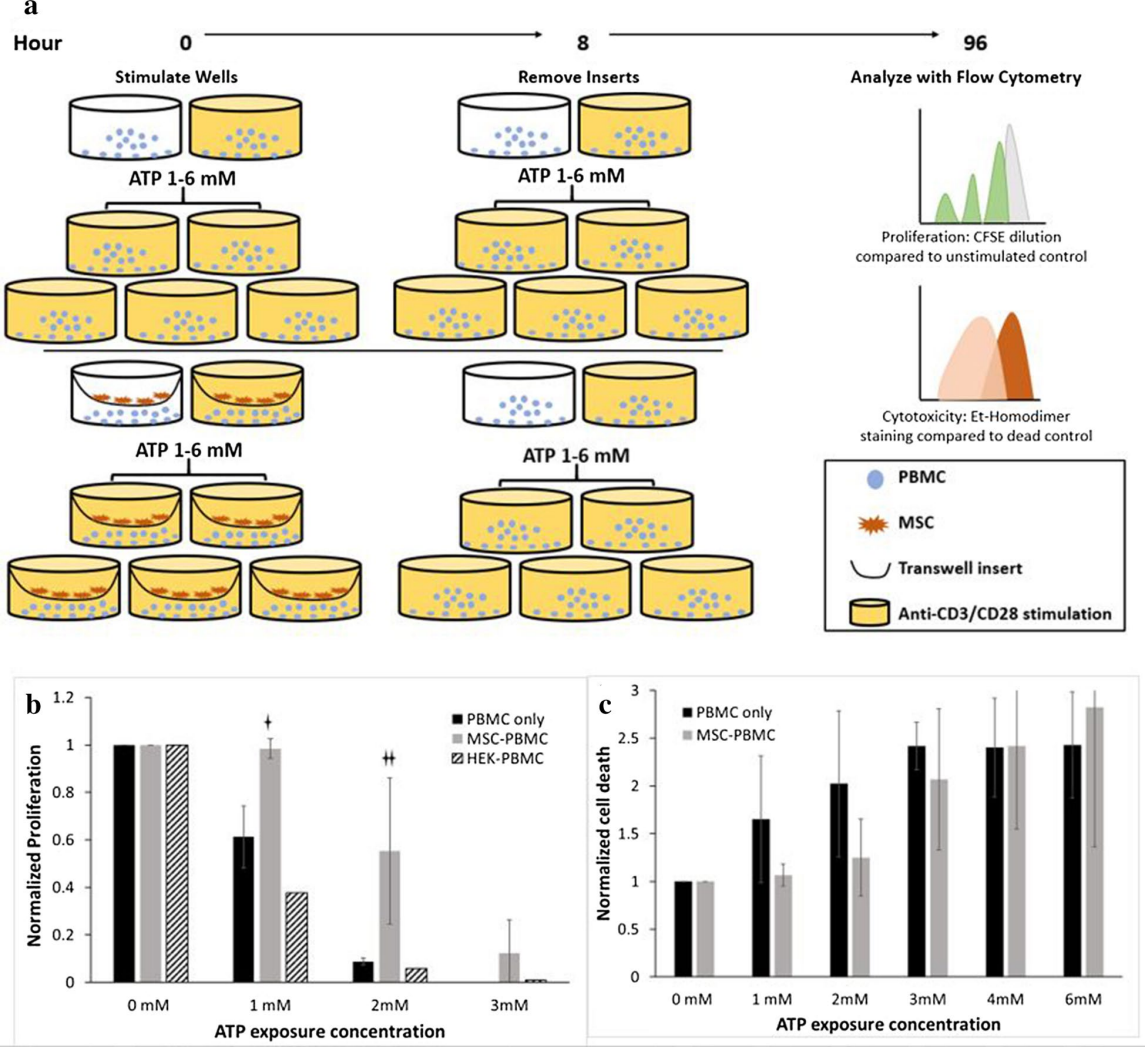
Lymphocyte response to ATP exposure. **a** PMBCs were cultured with or without MSCs during the first 8 h of stimulation and then allowed to grow until 96 h after co-stimulation. **b** The lymphocyte population of PBMCs was gated using flow cytometry and as ATP exposure increases, the proliferation of lymphocytes only decreases. At 1 mM exposure, proliferation is completely restored, and at 2 mM, MSCs allow for significantly more proliferation than the control. HEK cells which do not express the ecto-enzymes for ATP hydrolysis were included as a negative control. **c** At low levels of ATP exposure, cytotoxicity is lower in MSC co-cultured groups and as ATP dose increases, the 50,000 MSCs are insufficient in hydrolyzing the ATP enough to reduce cytotoxicity compared to the negative control. For both response experiments, n = 3

T-cell proliferation in response to mitogen or cytokine stimulation has been widely explored to show that MSCs are immunosuppressive by preventing T-cell proliferation, but mechanisms are unclear [18–20]. For lymphocytes alone, co-stimulation with anti-CD3/CD28 and ATP has been shown to induce a dose-dependent response: 250 nM is enough to activate CD4+ cell proliferation but 1 mM ATP induces apoptosis in these cells while inducing the greatest increase in proliferation of T-regs compared to lower doses [7]. Similarly, we stimulated lymphocytes with anti-CD3/CD28 antibodies and when T-cells are stimulated for 72 h or more, we see an increase in proliferation (data not shown), but when ATP is added, the proliferation compared to the unexposed control decreases at the concentration of ATP increases (Fig. 4b). For lymphocytes that are co-cultured with MSCs for 8 h and then left to be stimulated for 96 total hours, proliferation is restored compared to lymphocyte only wells (gray bars) as shown in Fig. 4b. This was confirmed further by co-culturing a cell type with the lymphocytes that does not express the ectoenzymes CD39 and CD73, HEK293 cells. In this group, the proliferation was similar to low levels seen in the lymphocyte only group. However, the limit for which MSCs can hydrolyze ATP becomes apparent at high concentrations of 3 mM where proliferation is significantly lower than both the 1- and 2-mM exposure group. Furthermore, proliferation in the MSC-PBMC group for both 1- and 2-mM doses was significantly higher than the PBMC only group. At a dose of 1 mM extracellular ATP, MSCs almost completely restore proliferation to levels seen by anti-CD3/CD28 stimulation alone. Coincidentally, the mean level of proliferation in the MSC-PBMC group exposed to 2 mM ATP is very similar to that of PBMC only exposed to 1 mM. This begins to show the limitation of how much ATP the MSCs can hydrolyze. Contrary to the results seen previously, co-stimulation with anti-CD3/CD28 antibodies and ATP decreases lymphocyte proliferation, which may because T-regs were not isolated, so this relatively high concentration of ATP for CD4+ T-cells would elicit apoptosis, but in the presence of MSCs, it allows CD4+ cells to persist and proliferate by ameliorating the cytotoxic effects of ATP.

### MSCs reduce lymphocyte death when exposed to increasing concentrations of ATP

Previous experimental studies have demonstrated that when CD4+ T-cells are exposed to high concentrations of extracellular ATP, apoptosis is induced, while T-regs are resistant to apoptosis at a 1 mM concentration [7]. To exhibit this phenomenon when MSCs are present to relieve the lymphocytes of ATP effects, we compared cell death of PBMC monocultures to PBMC-MSC co-cultures. In the monocultures, Fig. 4c shows that as concentration of ATP increases, cell death increases, but in the presence of MSCs, cell death is reduced. The effects of ATP exposure on lymphocyte cell death were greatest at concentrations of 3 mM, which in the lower range of what extracellular concentrations can reach in chronic inflammatory diseases. With the model, the predicted amount of ATP that 50,000 MSCs can hydrolyze in 8 h is 572 nmol, which in a 24-well plate is equivalent to 1 mM concentration. This is reinforced in the cytotoxicity result because at 1 mM (equivalent to 500 nmol in 0.5 mL), cytotoxicity in the MSC-PBMC group, while not statistically significant, is maintained around the control value. Additionally, cytotoxicity starts to increase at 2- and 3-mM concentrations in the MSC-PBMC group but not as high as PBMCs only. MSC protection from cell death in the presence of ATP is seen at doses which the MSCs can hydrolyze, but not at greater doses. Limiting exposure to only the first 8 h of the 96-h stimulation strengthens the findings that cell death observed is due to ATP exposure.

### MSCs reduce IL-1beta secretion from monocytes when exposed to ATP

**Fig. 5:**
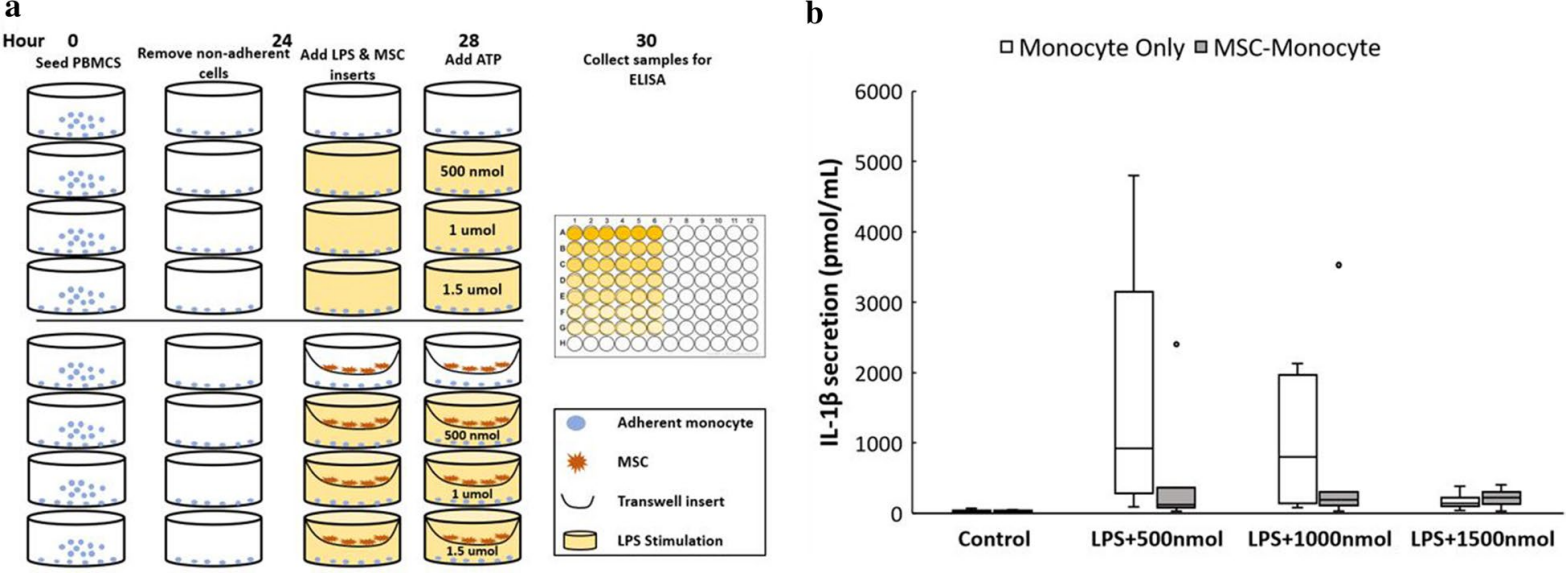
ELISA quantification of IL-1β secretion from monocytes when co-stimulated with LPS and ATP. **a** After culturing monocytes with or without MSCs in transwell inserts during the stimulation period, samples were analyzed with IL-1beta ELISA. **b** The cocultured groups released significantly less IL-1β than monocytes alone (n = 5 per group) where p < 0.10 for the 500 and 1000 nmol ATP groups. At these doses, the monocyte only secretion was significantly higher than the control, compared to 1500 nmol which was too high to elicit IL-1 β secretion

Co-stimulation of monocytes with lipopolysaccharide solution (LPS) and ATP induces proinflammatory IL-1β to be secreted. Previous studies have demonstrated a “bell-curve” response for ATP exposure ranging from 100 μM to 5 mM, which has been later confirmed as a result of active secretion rather than passive release [25]. Similarly, our results show that as the concentration of ATP increases, the effects on IL-1β become less apparent as shown in Fig. 5b. However, there are orders of magnitude more IL-1β secretion in LPS-primed monocytes exposed to 500 nmol compared to the control. To show the predictive power of the MSC hydrolysis models, monocytes were co-cultured with MSCs throughout 6 h of stimulation with LPS and ATP and IL-1β secretion was significantly suppressed compared to controls (p < 0.10). This was seen for both the 500 and 1000 nmol exposure groups, but 1500 nmol was too high to elicit IL-1β secretion from monocytes alone. Additionally, the mean secretion in MSC-monocyte groups when exposed to 1000 nmol is higher than in the 500 nmol, reinforcing the predictions from the model that 1000 nmol would be too high of a dose for MSCs to hydrolyze in that timespan.

## Discussion

### MSCs as a therapeutic for chronic inflammatory diseases

MSCs have potential in a wide range of indications where inflammation plays a role. Furthermore, ATP is a main contender in these indications and ameliorating the effects of ATP is a promising target through receptor antagonists [11]. As an alternative to blocking ATP receptors, MSCs provide an added benefit through their ability to convert ATP to immunosuppressive adenosine. In addition to ATP-related effects, MSCs have been shown to modulate inflammatory environments, secrete anti-inflammatory soluble factors, and home to injured areas. Here we have shown that in 2D in vitro systems, MSCs exemplify conversion of ATP to immunosuppressive adenosine, but also do so in a way that can be modeled by enzyme kinetics. While we primarily model the hydrolysis of ATP, quantified through an ATP based assay, we found that adenosine production is efficiently taking place at these rates as well. Furthermore, they can help restore immune function in cells that are affected by chronic stress caused by extracellular ATP. They do this in a multifaceted way depending on the cell type, where lymphocytes are protected from cytotoxic effects and allowed to respond through proliferation, while monocytes reduce secretion of proinflammatory IL-1β. There may be more effects produced by MSCs in the presence of ATP that are yet to be explored. Future experiments could investigate the effect of MSCs on immune cell expression of purinergic receptors and how that alters ATP hydrolysis rates.

### Translational power of kinetic model

Dosing for cell therapeutics remains a constant challenge. In showing that MSCs follow first-order enzyme kinetics for hydrolyzing ATP, we develop a predictive model to show the maximum amount of ATP a single MSC can hydrolyze. In the 2D systems, predicted rates were observed by exposing MSCs to excessive amounts of ATP and experimentally extracting hydrolysis at maximal rates. This was confirmed with adenosine production to match hydrolyzed ATP amounts as well. Further studies would need to be conducted in 3D model systems, such as bioreactors, to analyze how this model is applicable in vivo. It begins to develop the idea of ATP being a substrate rather than a side effect. Theoretically, this could be applied for other mechanisms related to MSCs, such as soluble factors. This model does not take into account the effect of MSCs on surrounding cells, as has been reported. To absolutely characterize the hydrolysis potential, the other cell types would need to be in co-culture during the hydrolysis time studies. A future study could be done to look at the rate of hydrolysis in the presence of monocytes or lymphocytes.

### Physiologic response to extracellular ATP

The dynamic response of MSCs has been exhibited through clinical trials, animal studies and in vitro assays with varying immune cells. Here we show an alternative to pharmaceutical approaches that block ATP receptors, and instead introduce a multipotent therapeutic that converts the ATP to adenosine. The effects of ATP on both lymphocytes and monocytes were shown to differ drastically in that monocytes are induced to secrete IL-1β up to a threshold, while lymphocytes, when co-stimulated with anti-CD3/CD28, are less proliferative and more cytotoxic at very high concentrations of ATP. In the case of monocytes, cells appeared to have a stimulation limit for each they would secrete IL-1β when co-stimulated with LPS and ATP are very high concentrations. However, for the ranges of ATP dosing that did elicit secretion, MSCs showed hydrolysis ability by converting the ATP to adenosine and therefore reducing the amount of IL-1β secreted by the monocytes after just 6 h of co-culture. This limit on ATP stimulation has been previously observed, but the exposure dose is shown to be inconsistent [26]. For the lymphocytes, MSCs have traditionally been used to show decreased lymphocyte proliferation over a timespan of days when stimulants such as IL-2, PHA, and anti-CD3/CD28 are used. However, here we show the dynamic nature of an immune response by showing that extracellular ATP suppressed proliferation, and with MSCs in co-culture to ameliorate effects of ATP, proliferation of lymphocytes is restored. The effect of ATP on lymphocytes has been previously studied to show that there is a threshold for varying populations to induce either apoptosis or proliferation, and here we looked at the lymphocyte population as a whole [7]. At high concentrations of ATP, cytotoxicity is high after 96 h of stimulation, but in the presence of MSCs for only the first 8 h, cell death is prevented significantly. Contrary to effects on monocytes, MSCs may help to allow lymphocytes to continue with a persistent immune response in the presence of extracellular ATP.

### Implications in MSC dosing

With a growing number of clinical trials using both allogeneic and autologous MSCs for cell therapy, in vivo persistence is a continuing challenge. Systemic infusions subjects MSCs to a number of stressors that reduce persistence such as shear stress, and nutrient transport limitations [27]. Despite this, MSCs have been shown to provide long-term benefits that extend well beyond their circulation time [28]. Here we show that in a matter of hours, MSCs effect lymphocyte proliferation and cell death days after MSCs were present. This may better model the therapeutic effect of MSCs with systemic infusion because it shows that in the short-term the benefit of MSCs may be acting to allow for lymphocyte proliferation during inflammation rather than suppress it. The transient nature of MSC T-cell suppression has been identified previously through T-cell responsiveness to allo-antigens after removal of MSC co-cultures [29]. Dosing is another challenge as well which with experimental modeling as described, may better help to bridge the gap between in vitro and in vivo data especially given that the timespan of our experiments are within range of MSC circulation times. Many studies have shown MSCs suppressing lymphocyte proliferation after days of co-culture but in showing that proliferation can be restored in the timespan that MSCs may persist, we give insight into more accurate dosing predictions [30–32]. Dosing for current clinical MSC therapies are on the order of 1 million cells/kg bodyweight for indications such as graft vs host disease [33–36]. With the model shown, we show that MSC dose can be predicted as a function of the amount of ATP present. For the lymphocyte experiments, it was calculated that 50,000 MSCs could hydrolyze a maximum of 572 nmol (1 mM for 24-well plates) of ATP in 8 h. The MSC-PBMC with 1 mM ATP group was the only one for both proliferation and cell death that maintained values similar to the control. At doses higher than this, 50,000 MSCs proves to be too little to restore proliferation or completely prevent cell death in lymphocytes. For a dose of 200 million cells delivered intravenously, theoretically, approximately 1 mmol of ATP could be hydrolyzed during 1 h. Based on the persistence of cells with this delivery method, it could be determined that this dose is too high or too low based on the amount of extracellular ATP present. To fully understand the dosing for translation in vivo, 3D culture systems could be used to adapt current models for accuracy in volumetric dosing.

## Conclusions

With cell-therapy dosing becoming more relevant as MSC therapies continue to emerge, there is a strong need to understand the mechanisms behind these multipotent cells. We show here that for a particular aspect of the inflammatory response, extracellular ATP, dosing can be quantified through experimental kinetic modeling in vitro. Through cell-based assays, the physiological response can be predicted using experimentally derived hydrolysis rates and a dynamic response is uncovered. The predicted outcomes of the functional cell assays were to suppress cytotoxicity, pro-inflammatory cytokine release and proliferation. Contrary to what was expected, we show that proliferation is restored in lymphocytes when co-stimulated with cytokines due to the short exposure time with MSC co-cultures. In all cases, the ability of MSCs to ameliorate the effects of extracellular ATP was exhibited in a dose-dependent manner. Further studies can be done to exploit the translational power of the model in 3D culture settings.

## Additional file

**Additional file 1: Figure S1.** MSCs convert ATP to Adenosine which can be inhibited by POM-1. (A) For MSCs dosed with 1 mM ATP (500 nmol), adenosine production increases with time. Hydrolyzed ATP and adenosine production were measured at each timepoint to show that 95% of ATP hydrolyzed by cells is converted to adenosine. The maximum amount of ATP hydrolysis predicted by models at this density is 120 nmol (when operating at Vmax) and experimentally we find 118 nmol is hydrolyzed and 112 nmol is converted to adenosine. (B) To inhibit CD39 activity, MSCs were treated for 20 min with 100 µM of POM-1 inhibitor and no measurable adenosine was recorded until 2 h after ATP treatment and remains significantly less than untreated groups. **Figure S2.** Fibroblast cells hydrolysis of ATP. (A) Expression of ectoenzymes CD73 and CD39 is similar to that of MSCs with normal dermal fibroblasts and pancreatic cancer-associated fibroblasts. CD73 is high expressed, while CD39 is weakly expressed. (B) When exposed to 64 nmol of ATP, both fibroblasts cell types can hydrolyze the ATP completely within 2 h.

## Data Availability

The data generated of analyzed during the current study are available from the corresponding author on reasonable request.

## Abbreviations

MSC: mesenchymal stromal cell
CD73: ecto-5′-nucleotidase ectonucleoside
CD39: triphosphate diphosphohydrolase-1
ATP: adenosine triphosphate
eATP: extracellular adenosine triphosphate
IL-1β: interleukin 1-beta
COPD: chronic obstructive pulmonary disease
LPS: lipopolysaccharide solution
P2R: purinergic receptor
P2X1: purinoreceptor 1
P2Y2: purinoreceptor 2
P2X7: purinoreceptor 7
CD4: cluster of differentiation 4
CD3: cluster of differentiation 3
CD28: cluster of differentiation 28
PBMC: peripheral blood mononuclear cells
FBS: fetal bovine serum
RPMI: Roswell Park Memorial Institute
PBS: phosphate buffered saline
FITC: fluorescein isothiocyanate
CFSE: carboxyfluorescein succinimidyl ester
Vmax: maximum velocity
Km: substrate concentration at half-maximum
HEK293: human embryonic kidney cell
ELISA: enzyme-linked immunosorbent assay
ANOVA: analysis of variance
PHA: phytohaemagglutinin
