# Supplementary figures and images for: Kinetics of MSC-based enzyme therapy for immunoregulation
Source: J Transl Med. 2019 Aug 13;17:263. doi: 10.1186/s12967-019-2000-6 (PMC6693124; doi:10.1186/s12967-019-2000-6)

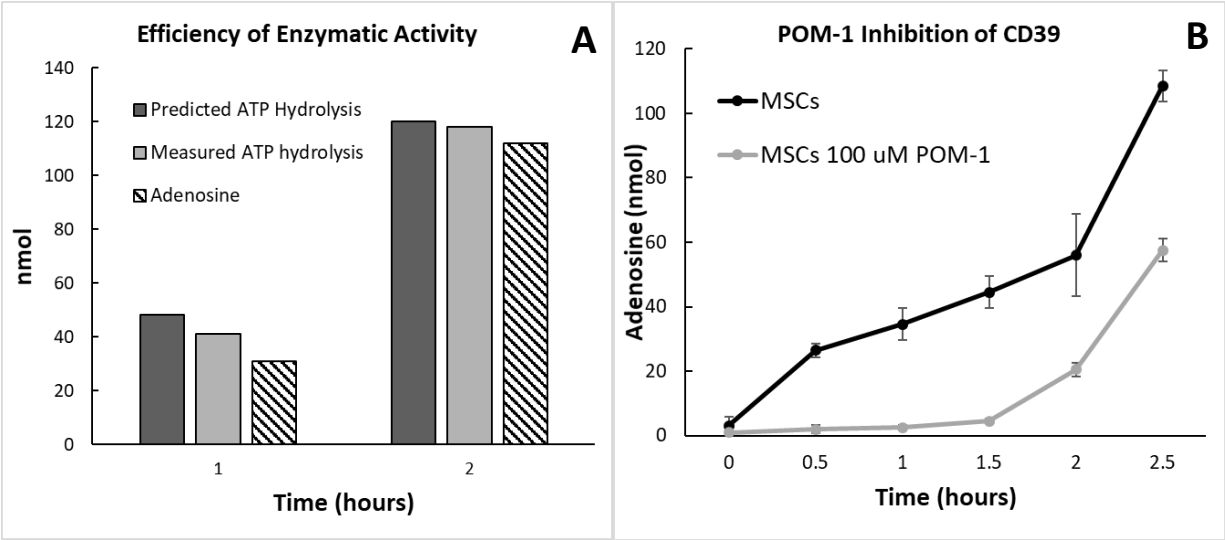

Figure S1

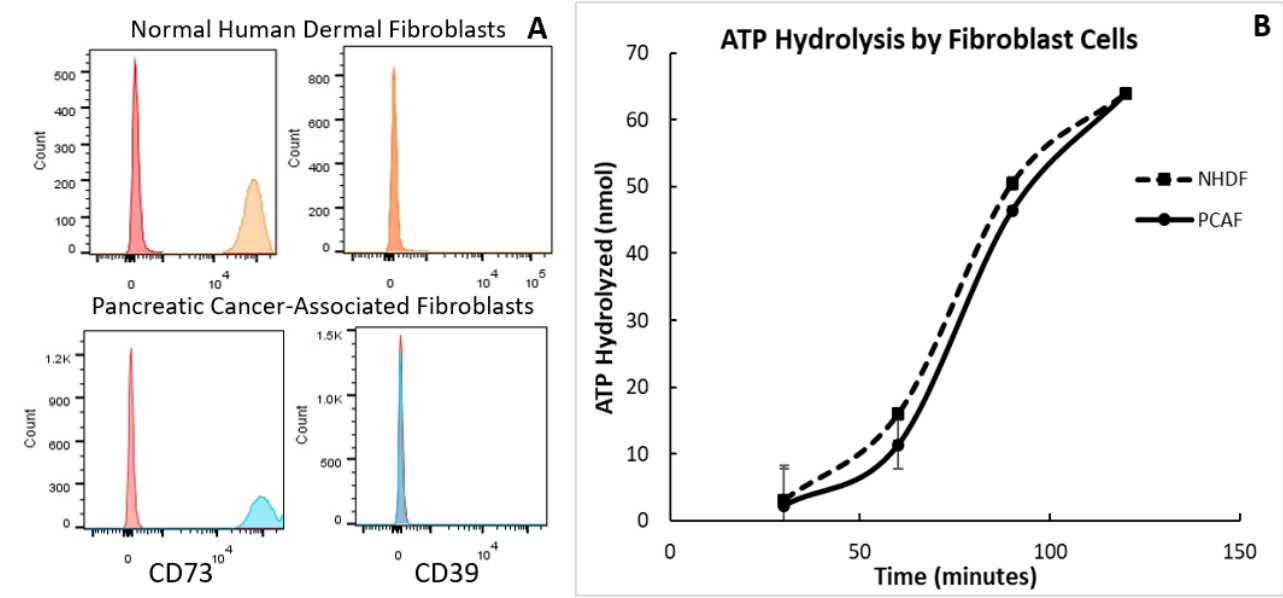

Figure S2

Supplement: Supplementary file 1 — Additional file 1: Figure S1. MSCs convert ATP to Adenosine which can be inhibited by POM-1. (A) For MSCs dosed with 1 mM ATP (500 nmol), adenosine production increases with time. Hydrolyzed ATP and adenosine production were measured at each timepoint to show that 95% of ATP hydrolyzed by cells is converted to adenosine. The maximum amount of ATP hydrolysis predicted by models at this density is 120 nmol (when operating at Vmax) and experimentally we find 118 nmol is hydrolyzed and 112 nmol is converted to adenosine. (B) To inhibit CD39 activity, MSCs were treated for 20 min with 100 µM of POM-1 inhibitor and no measurable adenosine was recorded until 2 h after ATP treatment and remains significantly less than untreated groups. Figure S2. Fibroblast cells hydrolysis of ATP. (A) Expression of ectoenzymes CD73 and CD39 is similar to that of MSCs with normal dermal fibroblasts and pancreatic cancer-associated fibroblasts. CD73 is high expressed, while CD39 is weakly expressed. (B) When exposed to 64 nmol of ATP, both fibroblasts cell types can hydrolyze the ATP completely within 2 h. [file 12967_2019_2000_MOESM1_ESM.pdf]
